# *Dentex maroccanus* Valenciennes, 1830 Otolith Morphology, Age, and Growth in the Aegean Sea (E. Mediterranean)

**DOI:** 10.3390/ani14213151

**Published:** 2024-11-02

**Authors:** Aglaia Legaki, Isabella Leonhard, Chryssi Mytilineou, Aikaterini Anastasopoulou

**Affiliations:** 1 Hellenic Centre for Marine Research, Institute of Marine Biological Resources and Inland Waters, P.O. Box 712, P.C. 19013 Anavyssos, Attiki, Greece; aglegaki@hcmr.gr (A.L.); chryssi@hcmr.gr (C.M.); 2 Institute of Palaeontology, University of Vienna, Josef-Holaubek-Platz 2, UZA II, 1090 Vienna, Austria; isabella.leonhard@univie.ac.at; 3 Institute of Evolutionary Biology, University of Warsaw, Miecznikowa 1, 02-089 Warsaw, Poland

**Keywords:** Morocco dentex, otolith morphometrics, growth parameters, aging, weight–length relationship

## Abstract

Otoliths are important structures for balance and hearing of fish and constitute a useful tool in fisheries science. This study provides, for the first time in the Mediterranean, information on the otolith morphometrics of *Dentex maroccanus*, collected from the South Aegean Sea, and enriches the existing information on its age and growth by sex. The otolith shape variables examined showed a more circular to square otolith shape, related to the body size. Significant differences between sexes were detected for the otolith *Area*, *Diameter*, *Perimeter,* and *Radius*. Exponential regressions were used to examine the relationship of the otolith variables with total body length, from which five showed a strong correlation (*Diameter*, *Width*, *Radius*, *Area,* and *Perimeter*). The eviscerated weight–length relationship exhibited an isometric growth for both sexes, whereas when total weight was applied, a positive allometric growth was found for females. Sagittal otolith readings revealed four age groups for females and five for males. A Bhattacharya method was used for age validation. Von Bertalanffy growth parameters were as follows: *L∞* = 23.08, *k* = 0.27, *t*_0_ = −1.93 for females and *L∞* = 24.07, *k* = 0.24, *t*_0_ = −2.26 for males. This research offers valuable biological information for *Dentex maroccanus* useful in fisheries science.

## Simple Summary

*Dentex maroccanus* otolith morphometrics and growth from the South Aegean Sea were studied using image analysis techniques. A circular or square shape can be assumed for the otoliths of the species in the Mediterranean, with small individuals presenting more circular-shaped otoliths than the larger ones. Differences between sexes were identified for some of the otolith parameters, while the growth parameters did not differ between them. Eviscerated, instead of total, weight is proposed to be used for the estimation of the weight–length relationship. The results of this study provide new information on the biological characteristics of this species in the Mediterranean Sea.

## 1. Introduction

Otoliths are calcified structures in the inner ear of fish that are used in various fields of biological, fisheries, and palaeontological studies, such as stock identification, age determination, taxonomy, ontogeny, phylogenetics, palaeoichthyology, determination of predator–prey interactions, and dietary studies (e.g., [1,2,3,4,5,6,7,8,9,10,11,12]). Otoliths continue to be a valuable tool currently since they offer a cost-effective and straightforward way to differentiate species and populations [13] as well as to study fish age and growth [14]. The study of the annual age and growth of fish is fundamental for the knowledge of growth rate in life history studies and stock assessment [14], information important in fisheries studies. The examination of otolith morphometrics can give information for paleodiversity, species separation, stock identification and discrimination, determination of population structure, as well as environmental and habitat variability [15,16,17,18,19]. Differences in otolith shape seem to be the result of the cooperative influence of genetics and environment [20,21,22,23], moderated through growth rate [6]. The shape indices represent the pattern of otolith growth in a two-dimensional surface [24]; in many species, otolith morphology varies from a round, circular shape in larval to an irregular, more elongated shape in adult fish [6,24]. Therefore, studying otolith morphology can inform about the evolutionary adaptations of species to their respective habitats and help in comprehending fish evolution and diversification [25], contributing in species identification [2], distinguishing stocks [26] and indicating how a fish adapts to environmental changes [27]. Lastly, the study and findings of sexual dimorphism in otolith shape of a species can offer information about possible different growth rates, distinct habitat usage, and sex-specific hormone levels between the two sexes [28,29,30,31,32].

The Morocco dentex, *Dentex maroccanus* Valenciennes, 1830, is a sparid demersal species. It is distributed in the Eastern Atlantic from the Bay of Biscay (occasionally further north) to the Gulf of Guinea, as well as in the southern, eastern, and central areas of the Mediterranean Sea [33,34,35]. The species occurs at depths ranging from 20 to 500 m, on various types of substrates, such as sand, gravel, or conglomerates [33,36]. It is a by-catch species of low economic value, which is mainly fished by trawl and artisanal fisheries [37]. Previous studies, conducted in the Aegean Sea, verified a relationship between this species distribution and environmental factors showing that salinity, temperature, and depth are the main drivers of its abundance [38,39].

Although there are some studies regarding the distribution, reproduction, population parameters, diet composition, and body morphometrics of *D. maroccanus* in the Mediterranean Sea [36,37,40,41,42,43,44,45], there is no information on the otolith morphometrics of this species. Age and growth by sex of the Morocco dentex in the Mediterranean Sea have been estimated in Tunisian and Algerian waters based on scales [46,47]. Moreover, two studies have been conducted in the North Aegean Sea based on otolith readings but for both sexes combined [48,49].

The objectives of the present work were as follows: (a) to investigate *D. maroccanus* otolith morphometrics for the first time in the Mediterranean and to examine possible differences in sagitta otolith shape between sexes, and (b) to improve our knowledge on the age and growth of this species and compare these biological characteristics with those from other areas. This information will be useful for biological and fisheries studies.

## 2. Materials and Methods

### 2.1. Study Area and Sampling

**Figure 1:**
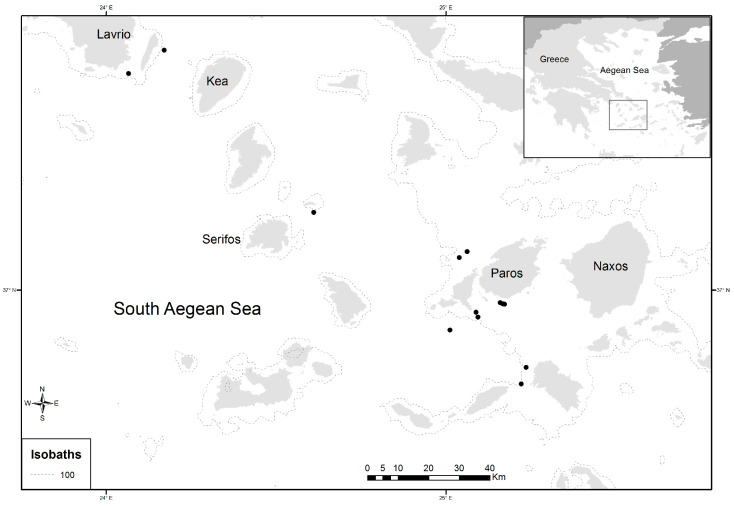
Map of study area and location of sampling sites (dots in black) of *Dentex maroccanus* in the South Aegean Sea.

In total, 405 individuals of *D. maroccanus* were collected during two experimental fishing surveys in September–October of 2014 and May–June of 2015. Sampling was performed at 13 stations (depths between 70 and 185 m) located in the South Aegean Sea (Figure 1), using bottom trawl nets. Individual total body length (*TL*, cm) and sex, identified by macroscopic examination of the gonads, were reported for each specimen. Their sizes ranged from 9.2 to 19.2 cm total length (*TL*). Left and right sagittal otoliths from each fish were extracted, rinsed with fresh water, and stored dry. Calibrated digital images of the right otolith were obtained under reflected light using a binocular stereoscope connected to the digital Image-Pro Plus software system (Version 4.5.0.29), which was also used to measure the otolith morphometric variables.

### 2.2. Data Analysis

#### 2.2.1. Length Frequency Distribution

The length (*TL*) frequency distribution of the *D. maroccanus* samples was calculated based on classes of 0.5 cm intervals for the study area.

#### 2.2.2. Otolith Morphometrics

**Figure 2:**
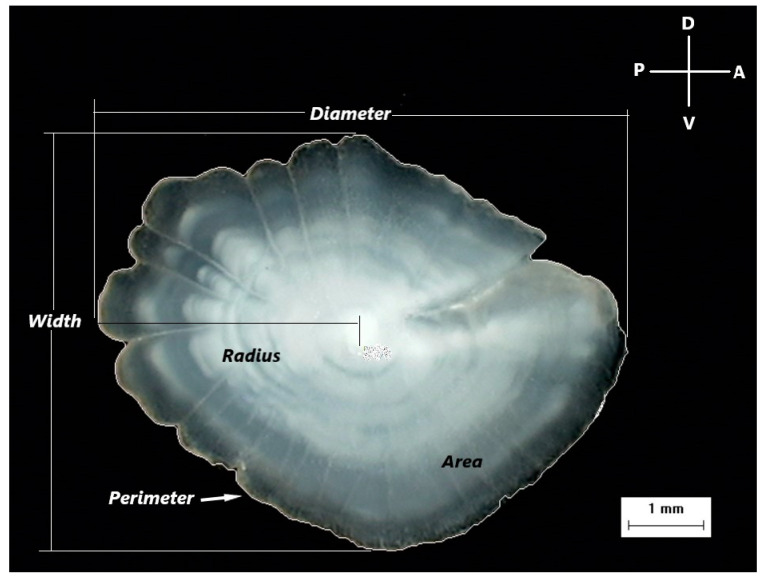
A digital image of the otolith of *Dentex maroccanus* from the South Aegean Sea, with the measured morphometric variables (*Diameter*, *Width*, *Radius*, *Area*, *Perimeter*), the orientation of the otolith (D: Dorsal, V: Ventral, P: Posterior, A: Anterior), and the scale of the image.

Due to the statistically significant difference (Kolmogorov–Smirnov test, *p* < 0.01) in the total body length frequency distribution between the two sexes, an approximately similar size range was used for the examined individuals to eliminate the effect of size on otolith variables. In total, 141 females and 143 males were used in the morphometric analysis, with length classes ranging between 92 and 181 mm and 96 and 186 mm, respectively. The new selected female and male samples did not show any statistically significant difference (Kolmogorov–Smirnov test, *p* = 0.051). Otolith morphometric measurements were carried out on the digital images of otoliths, excluding incomplete or damaged otoliths. The following otolith morphometric variables were examined: *Radius* (*R*, mm)—the longest distance from the otolith nucleus to the postrostrum; *Diameter* (*D*, mm)—the maximum distance between the rostrum and the postrostrum passing through the nucleus of the otolith; *Width* (*W*, mm)—the maximum distance between ventral and dorsal edges, a line perpendicular to Diameter; *Area* (*A*, mm^2^)—calculated for the largest surface area of the otolith; *Perimeter* (*P*, mm)—the length of the outline of the otolith using a polygonal outline; and *Roundness* (*Rn*)—the ratio between the actual area and the area of a circle of the same length (Figure 2).

Similarly, four additional shape indices (*C*: *Circularity*, *Rc*: *Rectangularity*, *FF*: *Form Factor,* and *E*: *Ellipticity*) were calculated using the following formulae [2,50]:

*C* = (Perimeter^2^/Area): *Circularity* compares otolith shape to a perfect circle showing values equal to 4π;

*Rc* = [Area/(Diameter × Width)]: *Rectangularity* describes the variations in the otolith length and width with respect to the area, with 1.0 indicating a perfect square;

*FF* = [(4π × Area)/Perimeter^2^]: *Form Factor* is a means to estimate the surface area irregularity, with a perfect circle taking values of 1.0;

*E* = [(Diameter − Width)/(Diameter + Width)]: *Ellipticity* indicates whether the changes in the axes are proportional.

The relationship of each morphometric variable with *TL* was examined for females and males separately applying the exponential regression (Y = a*TL^b^, where *Y* is the otolith variable in mm, *TL* is the Total Length in mm, *a* is the intercept, and *b* is the slope of the regression). For the shape variables of which negative relationship with *TL* was detected, a comparison between small and large individuals (of the two sexes combined) was performed with a one-way analysis of variance. The threshold between small and large samples was defined at the length of 130 mm.

Descriptive statistics (Mean ± Standard Error) for each morphometric variable of the *D. maroccanus* otoliths were applied. A multivariate GLM (General Linear Model) was assigned to test the effect of sex on otolith variables and identify the importance of each one of them to this effect. To ensure unbiased comparisons between sexes, the size-effect had to be removed from the variables with statistically significant relationship with *TL*, converting them to standardized values. For the standardization of the variables related with *TL,* the following procedures were performed: (i) analysis of covariance (ANCOVA) was used to identify if the slope *b* of the regression Y = a*TL^b^ was consistent between the two sexes, (ii) otolith variables which did not present consistent *b* between the two sexes were excluded from the analysis [51], and (iii) for the otolith variables with consistent *b*, the formula of Elliott et al. [52] was used to calculate the standardized values of the otolith variables: *M_s_ = M_o_(TL_s_/TL_o_)^b^*, where *M_s_* = standardized otolith variable, *M_o_* = measured otolith variable, *TL_s_* = overall (arithmetic) mean total length for all fish from all samples in each analysis, and *TL_o_* = total length of specimen, based on a common within-sex *b* for each measured otolith variable, estimated by a common exponential regression for both sexes. In the present study, the otolith variable *Width* was excluded from the GLM analysis because of a not-consistent *b* between the two sexes, which did not permit us to calculate the standardized values of this variable. Therefore, for the GLM analysis, the nine otolith variables (except *Width*) were used as dependent variables, while sex was used as independent. Because of the detection of non-normality, log, square root, and 1/x transformation were used when needed.

#### 2.2.3. Growth and Age

The weight–length relationship (WLR) was estimated for data from 185 females and 220 males. The power function W = a*TL^b^ was used to estimate the relationship between *TL* (in cm) and Total Weight *(TW) or* Eviscerated Weight (*EW*) (in gr), where *a* is the intercept, and *b* is the slope of the regression. Both forms of weight were used to test the effect on the growth type (isometry/allometry). For each sex, the null hypothesis for isometric growth (Ho: *b* = 3) was tested by using Student’s *t*-test. Comparison of the weight–length regressions for the two sexes was performed using analysis of covariance. Differences were considered at the significant level of *a* = 0.05.

For age estimation, 185 females and 220 males were used. Hyaline growth rings, which are formed once a year and represent the time of reduced growth, were counted and measured from the nucleus to the post-rostrum of the right otolith’s distal surface. Age estimation was conducted by two readers. Age length keys were produced for females and males. Growth parameters of the Von Bertalanffy growth equation were estimated by sex, based on this Equation: *Lt = L∞ (1 − e^−K(t−t*_0_*^^)^)*, where *Lt* is the predicted length at age t in cm, *L∞* is the asymptotic length expressing the mean length that the fish would reach if it would grow indefinitely in cm, *k* is the growth coefficient which determines the rate at which the fish approaches *L∞* in year^−1^, and *t*_0_ is the hypothetical age at length zero in years. Growth parameters between sexes were tested using Student’s *t*-test. The growth performance index *Φ′ = Log (K) + 2Log (L∞)* [53] was estimated for males and females, separately.

Age validation was based on the Bhattacharya method (1967) [54], as applied by FiSAT II (version 1.2.2 computer program) [55]. The method was used to discriminate normal distributions that correspond to age groups.

## 3. Results

### 3.1. Length Distribution

**Figure 3:**
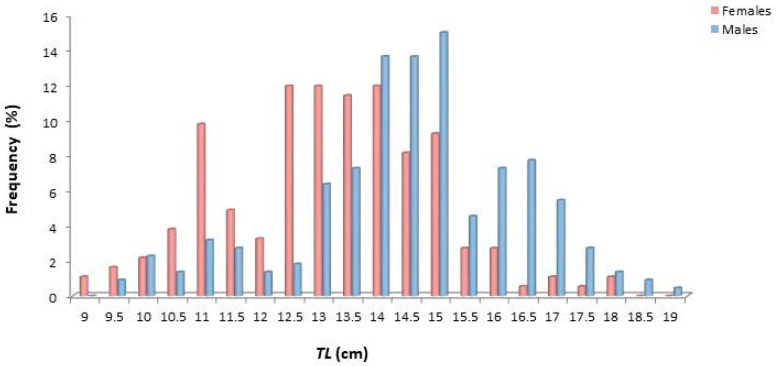
Length frequency distribution (*TL*, cm) of female and male *Dentex maroccanus* in the South Aegean Sea.

The size of females varied from 9.2 to 18.1 cm *TL*, while that of males from 9.6 to 19.2 cm (Figure 3). Males showed dominance towards greater sizes, while females were generally smaller in size.

### 3.2. Otolith Morphometrics

The results of the exponential relationship between each otolith variable and *TL* for females and males separately are shown in Table 1. These relationships were statistically significant, except for *Rectangularity* (*Rc*) (in both sexes) and *Ellipticity* (*E*) (in females) (*p*-value > 0.05). The *Diameter* (*D*), *Width* (*W*), *Radius* (*R*), *Area* (*A*), and *Perimeter* (*P*) of the otolith showed a strong positive relationship with *TL*. The values of the shape indices *Roundness* (*Rn*), *Circularity* (*C*), and *Form Factor* (*FF*) showed a statistically significant but weak correlation with *TL*. Comparison of regression lines for each otolith variable between females and males showed differences for the variable of *Width* (*W*) (*p*-value = 0.02), while for the other examined variables, no differences were detected (*p*-value > 0.05). Further examination of the negative relationship found for Roundness (*Rn*) and *Circularity* (*C*) with *TL*, using ANOVA, showed a statistically significant difference between small and larger individuals (*p*-value < 0.05), with mean values being higher in smaller individuals than the larger ones.

The descriptive statistics of the otolith morphometric variables by sex are given in Table 2. Females presented higher standardized values of the otolith variables than males. For both sexes, *Circularity* (*C*) presented mean value > 4π, those for the *Form Factor* (*FF*) were close to 1, while that for *Rectangularity* (*Rc*) was less than 1, and that for *Ellipticity* (*E*) was quite lower than 1 (Table 2).

The multivariate GLM, which has taken into consideration all the variables (except *Width* as explained in M&M), showed statistically significant relationships between four otolith variables (*A*, *D*, *P*, *R*) and sex, with, more importantly, the otolith *Area,* followed by the otolith *Diameter* (Table 3).

### 3.3. Growth and Age

The weight–length relationship parameters for females and males are given in Table 4. In case of *TW* (Table 4a), the value of *b* for males did not differ significantly from 3, which indicates isometric growth (*t*-test = 1.33; *p*-value = 0.18). For females, *b* differed significantly from 3, which means that females exhibited allometric growth (*t*-test = 3.51; *p*-value = 0.0005). However, when *EW* (Table 4b) was used for the estimation of the relationship between weight and length, the value of *b* did not differ significantly from 3 for both sexes (females *t*-test = 1.84, *p*-value = 0.067; males *t*-test = 1.66, *p*-value = 0.098), which indicates isometric growth. The comparison of the slope between sexes did not show a statistically significant difference in both cases (TW: *p*-value = 0.0895; EW: *p*-value = 0.7056).

**Figure 4:**
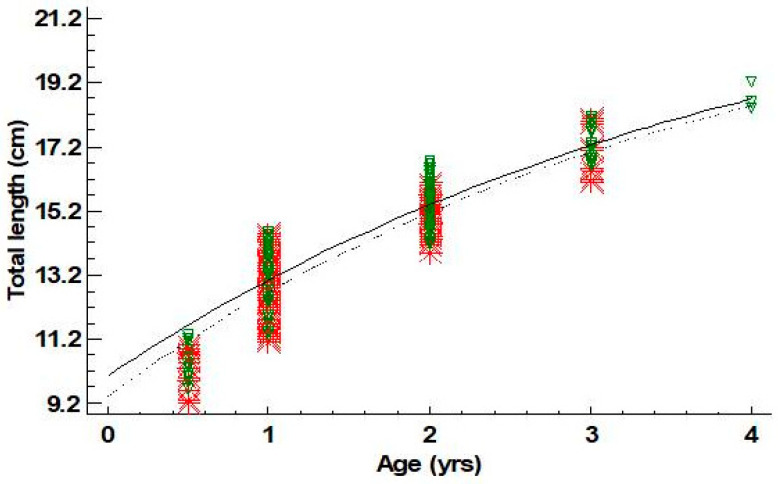
Von Bertalanffy growth curves fitted for females (- -*- -) and males (─∇─) of *Dentex maroccanus* in the South Aegean Sea (red asterisks: female observations—green triangles: male observations).

Estimated ages for males ranged between 0+ and 4 years old; those of females ranged between 0+ and 3. For males, the age group 2 was dominant (43.6%), while for females, the age group 1 was the one prevailing (58.4%) (Table 5). Four and five groups (cohorts) could be defined using the Bhattacharya method from length frequency data for females and males, respectively (Table 6). The mean lengths of these cohorts were quite similar to the mean lengths derived from the age–length key for the age groups I–III for females and I–IV for males (Table 6). Von Bertalanffy growth curves are shown in Figure 4. Growth parameters and growth performance index *Φ′* for females and males are presented in Table 7. Statistically significant differences were not found for *L∞* and *k* between sexes (*L∞*: *p*-value = 0.85; *k*: *p*-value = 0.82).

## 4. Discussion

In the current study, the age and growth of *D. maroccanus* were studied for the first time in the South Aegean Sea, contributing updated information to the biology of the species in the Mediterranean. Furthermore, for the first time in the Mediterranean, the morphological features of sagittal otoliths of *D. maroccanus* were analyzed in order to examine the otolith shape and their relationship with body size, offering information that might be used in the future in other comparative studies of the species from different areas. Finally, differences in the otolith morphology between sexes were examined for the first time in the Mediterranean.

Shape indices studied indicated that the otoliths of the Morocco dentex have a more circular-to-square shape because of the quite high estimated values of *Roundness*, *Form factor,* and *Rectangularity*. A more pentagonal shape for the otoliths of the species from the central-eastern Atlantic was assumed by Tuset et al. [15]. Regarding *Circularity*, the mean value by sex estimated in the present study was within the range of values reported by the above-mentioned researchers. However, this was not the case for *Rectangularity*, indicating that the otolith shape of the species in the Mediterranean is squarer than that of the Atlantic. Otolith shape is known to be influenced by ontogenetic [9], as well as environmental abiotic factors, such as water temperature [23,58]. Therefore, the differences identified between the current study and the one of Tuset et al. [15] might be due to the different fish size range of the examined samples (larger specimens in the Atlantic Ocean: 14.9–28.5 cm, than the specimens of the current study: 9.2–19.2 cm) or to the different environmental conditions between the two study areas.

In the present study, higher values of the standardized morphometric variables *Diameter*, *Radius*, *Area*, and *Perimeter* were found for females than males, with more pronounced differences for *A* and *D.* However, no significant sex differences were detected regarding the otolith shape. Different otolith growth patterns between sexes have been described for various species (e.g., *Prionotus nudigula*—[59], *Porichthys notatus*—[60]). No sex-specific differences regarding the otolith shape were also reported for other species such as *Gadus morhua* [21] and *Xiphias gladius* [61]. Variability in otolith morphometrics between the sexes has been associated with differences in environmental factors, distinct habitat usage, growth rate, sex-specific hormone levels, and reproductive behavior [1,10,28,29,30,31,32]. The shape and size of the sagittal may affect hearing capacities [62], and it is believed that large sagittae increase hearing sensitivity [63], which seems to be a more important attribute in benthic than pelagic species [22]. However, Kever et al. [62] found no direct link between otolith size and hearing capacities of the two ophidiiform species studied. Interestingly, Popper et al. [64] suggested that it is more likely that the selective pressures on otolith size and ear function are more related to the response to the rapid motions of the animals rather than to hearing. Lastly, diet composition has been related to otolith shape [65]. The differences identified in the present study between the otoliths of male and female *D. maroccanus* may be related to the hearing and/or swimming abilities and/or diet preferences of the two sexes. In fact, in eastern Algeria, differences in the feeding habits of the species were reported between the two sexes [42]. No literature was found regarding the hearing and swimming abilities of the species.

The exponential regression model was found to be more appropriate than the linear to describe the relationship between fish length and otolith variables, as also found in other relevant studies [66,67]. The negative relationship of *Roundness* and *Circularity* with total length indicated that the otoliths of *D. maroccanus* become less circular with increased body size. This better explains the otolith pentagonal shape mentioned by Tuset et al. [15], who examined larger individuals than those of this study. The equations between the otolith variables and total length are particularly useful for further otolith-size predictions (e.g., dietary studies), paleontological studies related to back-calculations of fish size and growth patterns, and ontogenetic studies related to otolith shape [68].

The examination of the weight–length relationship based on the eviscerated weight showed an isometric growth for female *D. maroccanus*, while the use of total weight revealed positive allometry. Taking into consideration that the spawning period of *D. maroccanus* in the Mediterranean has been recorded in summer and autumn [43,47,48] and that the sampling of the present study took place within this period, the two different types of growth found in females could be attributed to the weight of mature gonads affecting their total weight and finally the total weight–length relationship. Therefore, the use of eviscerated weight is recommended for the WLR study. López-Pérez et al. [69] suggest the use of eviscerated dry weight, since it more accurately reflects muscular growth, irrespective of trophic behavior (full or empty guts) or gonadal weight (important at maturation). Weight–length relationship studies for *D. maroccanus* from other Mediterranean areas showed variability expressed as positive or negative allometry and isometry [46,47,48,49,70]. Such differences in the parameter *b* of the WLR could be attributed to factors such as the sampling area, the sampling season, the life-history stage of the species, or the nutritional state of each individual [70,71], but they could also be related to the use of total or eviscerated weight, as proved in our study.

In the present study, the growth parameters of *D. maroccanus* did not differ between females and males, which implies that the two sexes approach their asymptotic length at a similar rate. The asymptotic maximum length for both sexes combined (23.14 cm *TL*) was the lowest among those reported by other studies conducted in the Mediterranean Sea (Table 7). In general, it seems that the *L∞* values from the Atlantic and the southwestern Mediterranean were higher than those from the eastern Mediterranean. This could be attributed to the lower nutrient contents in the latter area compared to the western Mediterranean Sea. Similarly, the growth performance index *Φ′* of *D. maroccanus* revealed the lowest values in the eastern basin of the Mediterranean Sea, while the highest value (2.45) has been reported from Algeria [47]. It could also be noticed that the *Φ′* values reported in the studies from various areas of the Aegean Sea, including those of the present work (Table 7), are quite similar, which may indicate a connectivity among the populations of the species in this region.

The present work provided important information concerning otolith morphometrics and age and growth of *D. maroccanus*, as well as existing differences between males and females. Further studies should reveal the potential effect of environment on these features and the link between the physiological and behavioral characteristics in them.

## 5. Conclusions

The results of the present study showed differences in the otolith morphometry of *D. maroccanus* between the two sexes and differences in the species growth pattern between eastern and western Mediterranean. Otolith morphometry, as well as age and growth, can be used to answer a variety of ecological and physiological questions. However, the complexity concerning otoliths and its implications for biological processes do not allow for a single-factor explanation.

## Figures and Tables

**Table 1:** Exponential regression between various otolith morphometric variables and fish total length (*TL*) of *Dentex maroccanus* for females and males: intercept value (*a*), regression slope (*b*), coefficient of determination (*R*^2^), correlation coefficient (*r*), and *: significant relation (*p*-value < 0.05); *D*: *Diameter*, *W*: *Width*, *R*: *Radius*, *A*: *Area*, *P*: *Perimeter*, *Rn*: *Roundness*, *C*: *Circularity*, *Rc*: *Rectangularity*, *FF*: *Form Factor*, *E*: *Ellipticity*. The *p*-value of the regressions and the *p*-value of the comparison of the slope *b* of the regression lines between sexes (ANCOVA) are also given.

Females						Males					
Relation	*a*	*b*	*r*	*R* ^2^	*p*-Value	*a*	*b*	*r*	*R* ^2^	*p*-Value	ANCOVA *p*-Value for *b*
***D-TL***	0.08	0.89	0.94	0.89	<0.05 *	0.07	0.92	0.95	0.91	<0.05 *	0.34
***W-TL***	0.05	0.93	0.96	0.92	<0.05 *	0.03	1.01	0.95	0.90	<0.05 *	**0.02**
***R-TL***	0.04	0.89	0.94	0.89	<0.05 *	0.04	0.93	0.95	0.91	<0.05 *	0.22
***A-TL***	0.003	1.83	0.96	0.92	<0.05 *	0.002	1.95	0.95	0.91	<0.05 *	0.08
***P-TL***	0.30	0.84	0.94	0.89	<0.05 *	0.21	0.91	0.94	0.88	<0.05 *	0.06
***Rn-TL***	2.27	−0.13	−0.43	0.19	<0.05 *	2.16	−0.12	−0.36	0.14	<0.05 *	0.75
***C-TL***	28.57	−0.13	−0.43	0.19	<0.05 *	27.18	−0.12	−0.36	0.13	<0.05 *	0.75
***Rc-TL***	0.71	0.02	0.13	0.02	>0.05	0.76	0.007	0.06	0.003	>0.05	-
***FF-TL***	0.44	0.13	0.43	0.19	<0.05 *	0.47	0.12	0.36	0.13	<0.05 *	0.75
***E-TL***	0.19	−0.03	−0.04	0.002	>0.05	0.62	−0.27	−0.36	0.13	<0.05 *	-

**Table 2:** Mean ± Standard Error (SE) of the otolith morphometric variables for females and males of *Dentex maroccanus* in the South Aegean Sea. The standardized values are also given (*italics*). *D*: *Diameter*, *W*: *Width*, *R*: *Radius*, *A*: *Area*, *P*: *Perimeter*, *Rn*: *Roundness*, *C*: *Circularity*, *Rc*: *Rectangularity*, *FF*: *Form factor*, *E*: *Ellipticity*. The common *b* value used for the estimation of their standardized values is also presented for the parameters significantly correlated with total length (*TL*).

Otolith Parameter	Mean ± SE		Common *b* of Exponential Regression
	Females	Males	
***D***	6.551 ± 0.069 *(6.667 ± 0.024)*	6.690 ± 0.070 *(6.587 ± 0.022)*	0.8995
***W***	4.661 ± 0.051	4.797 ± 0.055	-
***R***	3.394 ± 0.036 *(3.455 ± 0.013)*	3.480 ± 0.037 *(3.426 ± 0.012)*	0.9054
***A***	24.347 ± 0.503 *(24.834 ± 0.163)*	25.557 ± 0.535 *(24.347 ± 0.170)*	1.8798
***P***	18.834 ± 0.191 *(19.167 ± 0.067)*	19.296 ± 0.205 *(19.006 ± 0.075)*	0.8721
***Rn***	1.180 ± 0.004 *(1.175 ± 0.004)*	1.182 ± 0.005 *(1.183 ± 0.004)*	−0.1254
***C***	14.825 ± 0.054 *(14.771 ± 0.049)*	14.852 ± 0.057 *(14.867 ± 0.053)*	−0.1253
***Rc***	0.785 ± 0.001	0.783 ± 0.001	-
***FF***	0.849 ± 0.003 *(0.852 ± 0.003)*	0.847 ± 0.003 *(0.847 ± 0.003)*	0.1253
***E***	0.169 ± 0.001	0.168 ± 0.001	-

**Table 3:** Results of multivariate GLM analysis showing the effect of sex on each otolith variable of *Dentex maroccanus*. SS: Sum of Squares; df: degrees of freedom; MS: Mean Square; *D*: *Diameter*, LOG *R*: logarithm of *Radius*, LOG *A*: logarithm of *Area*, LOG *P*: logarithm of *Perimeter*, *Rn*: *Roundness*, *C*: *Circularity*, *Rc*: *Rectangularity*, SQRT *FF*: Square root of *Form factor*, *E*: *Ellipticity*.

Otolith Parameter	Type III SS	df	MS	F	*p*-Value
***D***	0.40	1	0.40	5.56	0.019 *
**LOG *R***	0.006	1	0.006	3.92	0.049 *
**LOG *A***	0.05	1	0.05	8.06	0.005 *
**LOG *P***	0.008	1	0.008	4.62	0.032 *
**1*/Rn***	0.001	1	0.0009	0.75	0.387
**1*/C***	0.00001	1	0.00001	0.74	0.391
***Rc***	0.0005	1	0.0005	2.03	0.156
**SQRT *FF***	0.0003	1	0.0003	0.75	0.386
***E***	0.0001	1	0.0001	0.49	0.487

*: significance level *a* = 0.05.

**Table 4:** (**a: top, b: bottom**) Weight–Length relationship parameters of *Dentex maroccanus* in the South Aegean Sea by sex; total length (*TL*) in cm, total weight (*TW*) in gr, eviscerated weight (*EW*) in gr, regression intercept (*a*), regression slope (*b*), correlation coefficient (*r*), coefficient of determination (*R*^2^). *p*-value at significance level *α* = 0.05.

**Sex**	***TL*** **(cm)**	***TW*** **(gr)**	***a***	***b***	***r***	***R*** **^2^**	***p*** **-Value**
**Females**	9.2–18.1	13–95	0.009	3.20	0.97	0.95	**<0.05**
**Males**	9.6–19.2	13–99.9	0.013	3.07	0.97	0.95	**<0.05**
**Sex**	***TL*** **(cm)**	***EW*** **(gr)**	***a***	***b***	***r***	***R*** **^2^**	***p*** **-Value**
**Females**	9.2–18.1	12.3–87.4	0.011	3.11	0.97	0.94	**<0.05**
**Males**	9.6–19.2	12.1–96.5	0.012	3.08	0.98	0.95	**<0.05**

**Table 5:** Age–length key of *Dentex maroccanus* in South Aegean Sea by sex (N: Sample size, %: Percentage of sample, MTL: Mean Total Length in cm, SE: Standard Error).

	Age (years)								
	Females				Males				
Length Interval (cm)	0+	I	II	III	0+	I	II	III	IV
**9.0–9.9**	5				2				
**10.0–10.9**	11				9				
**11.0–11.9**	6	21			5	9			
**12.0–12.9**		29				7			
**13.0–13.9**		40	3			30			
**14.0–14.9**		18	19			27	33		
**15.0–15.9**			22				41		
**16.0–16.9**			2	4			22	11	
**17.0–17.9**				3				18	
**18.0–18.9**				2				3	2
**19.0–19.9**									1
**N**	22	108	46	9	16	73	96	32	3
**%**	11.89	58.37	24.87	4.87	7.27	33.18	43.64	14.55	1.36
**MTL**	10.41	12.94	14.92	17.06	10.53	13.43	15.31	17.21	18.73
**SE**	0.12	0.09	0.08	0.27	0.14	0.1	0.07	0.08	0.24

**Table 6:** Computed mean length-at-age (cm) separated by Bhattacharya method (FiSAT program) for female and male *Dentex maroccanus* in South Aegean Sea (S.D.: Standard Deviation; S.I.: Separation Index).

Age Groups	Computed Mean Length (cm)	S.D.	S.I.
**Females**			
0+	11.21	0.63	n.a.
I	13.32	0.46	2.17
II	14.92	0.78	2.05
III	17.0	0.64	2.08
**Males**			
0+	11.14	0.93	n.a.
I	13.55	0.40	2.18
II	14.77	0.60	2.03
III	16.61	0.91	2.04
IV	18.50	1.0	2.0

**Table 7:** Von Bertalanffy growth parameters and growth performance index of *Dentex maroccanus* from different areas of the Atlantic and Mediterranean Sea [*L∞*: asymptotic length (cm), *k*: growth coefficient (yrs^−1^), *t*_0_: hypothetical age at length zero (yrs), *Φ′*: growth performance index].

Source	Area	Method	Size (cm)	Age (yrs)	Sex	*L*∞ (cm)	*k* (yrs^−1^)	*t*_0_ (yrs)	*Φ′*
	**Atlantic**								
**[56]**	White Cape (NE Atlantic)	Otoliths			C	34.3	0.180	−0.490	2.32
	Cape Verde (NE Atlantic)				C	32.5	0.180	−0.620	2.27
**[57]**	Southern Morocco (NE Atlantic)	Scales			C	30.34	0.191	−1.638	2.25
					M	30.24	0.164	−1.974	2.18
					F	31.55	0.181	−1.853	2.26
	**Mediterranean Sea**								
**[46]**	Tunisian coasts (SW Mediterranean)	Scales		1–7	C	33.54	0.191	−1.434	2.33
				1–7	M	33.89	0.184	−1.592	2.32
				1–7	F	35.93	0.156	−1.848	2.29
**[47]**	Eastern Algeria (SW Mediterranean)	Scales		1–8	C	37.26	0.2	0.5	2.45
			11.5–26.1	1–8	M	37.26	0.2	0.5	2.45
			10.5–26.7	1–7	F	36.64	0.2	0.5	2.36
**[48]**	Saros Bay (NE Mediterranean)	Otoliths	9.0– 22.0	1–3	C	25.31	0.49	−0.30	2.16
**[49]**	Izmir Bay (NE Mediterranean)	Otoliths	7.0–22.1	1–5	C	27.43	0.21	−0.68	2.20
**Present study**	South Aegean Sea (NE Mediterranean)	Otoliths	9.2–19.2	1–4	C	23.14	0.275	−1.94	2.17
			9.6–19.2	1–4	M	24.07	0.239	−2.26	2.14
			9.2–18.1	1–3	F	23.08	0.272	−1.93	2.16

## Data Availability

The data that support the findings of this study are available from the corresponding author upon reasonable request.
